# Supplementary material for: A ROR1 Small Molecule Inhibitor (KAN0441571C) Induced Significant Apoptosis of Mantle Cell Lymphoma (MCL) Cells
Source: Pharmaceutics. 2022 Oct 20;14(10):2238. doi: 10.3390/pharmaceutics14102238 (PMC9607197; doi:10.3390/pharmaceutics14102238)
Supplement: Supplementary file 1 [file pharmaceutics-14-02238-s001.zip › pharmaceutics-1903254-supplementary.pdf]

pharmaceutics

MDPI

Supplementary Materials: The following supporting information can be downloaded at: www.mdpi.com/xxx/s1

Supplementary Figure S1: Dose-dependent apoptosis of KAN0441571C. Analysis of apoptosis in primary MCL cells from a patient using Annexin V/PI staining in CD45$^+$/ROR1$^+$/CD3$^-$ gated cells after incubation for 24 h with KAN0441571C (25-2000 nM).

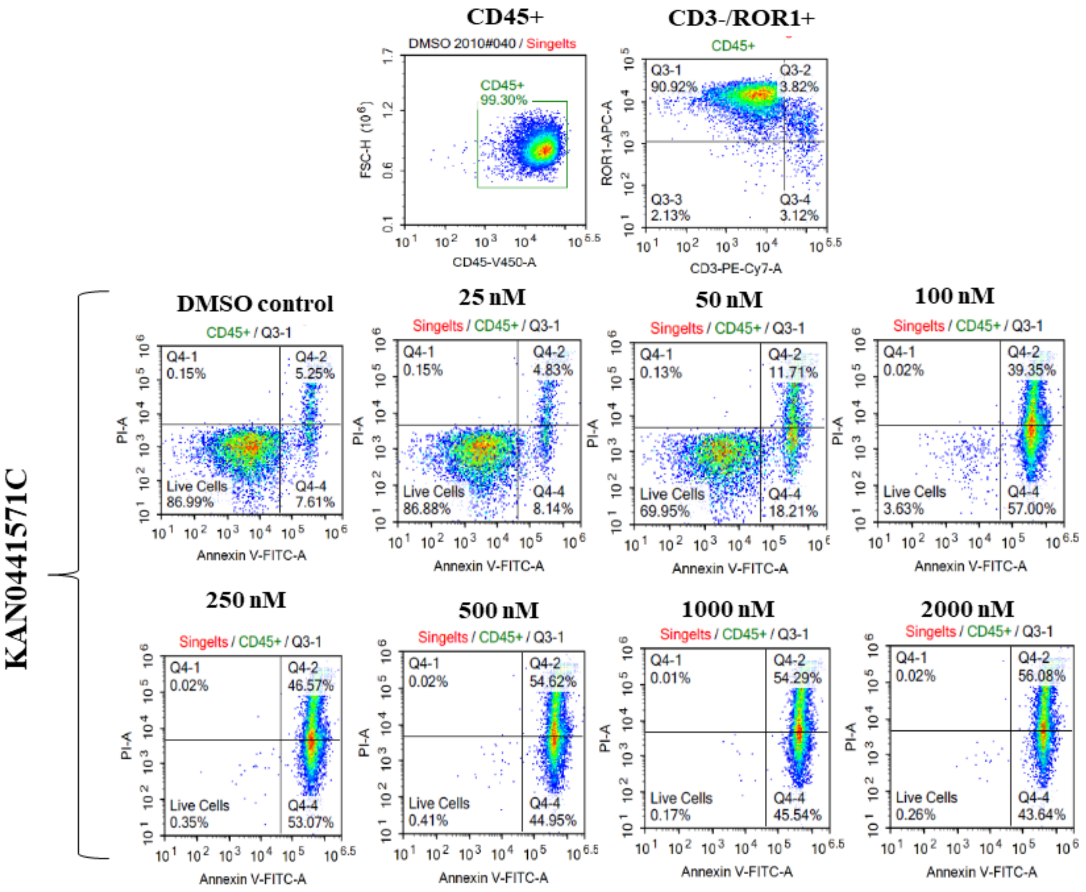

Supplementary Figure S2: Analysis of ROR1 expression in MCL cell lines. Analysis of ROR1 surface expression in 5 MCL cell lines (A). Western Blot analysis of total (t) ROR1 and phosphorylated (p) ROR1 proteins in 5 MCL cell lines before and after incubation (4 h) with the

ROR1 inhibitor, KAN044157C (250 and 500 nM). pROR1/ROR1 density has been shown under each blot (B).

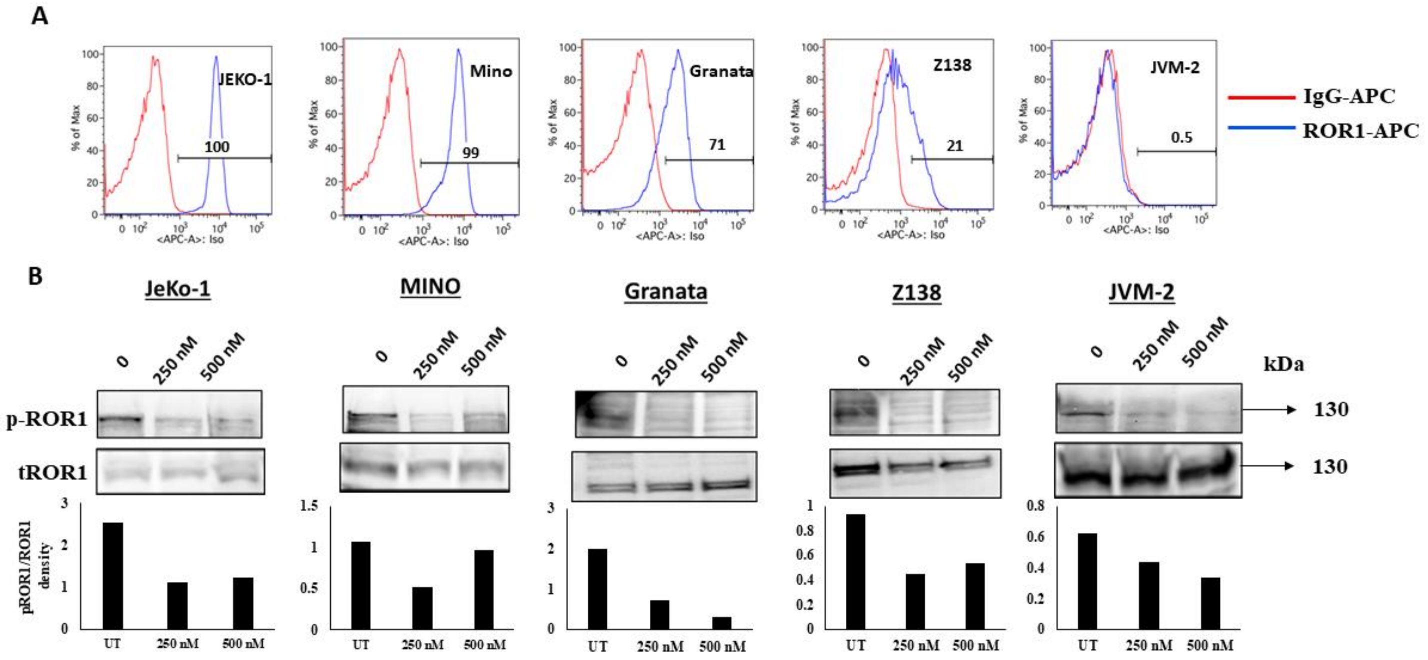

Supplementary Figure S3: MTT assay on 5 MCL cell lines. Cytotoxicity (MTT) in 5 MCL cell lines incubated for 72 h with EC_{50} concentration of KAN0441571C and equimolar concentration of venetoclax, ibrutinib, and bendamustine respectively. P-values comparing cytotoxicity of KAN0441571C alone against the combination of KAN0441571C with other drugs are shown at the top. ** $p < 0.01$, **** $p < 0.0001$; ns: not significant. Mann–Whitney U test was used for comparisons.

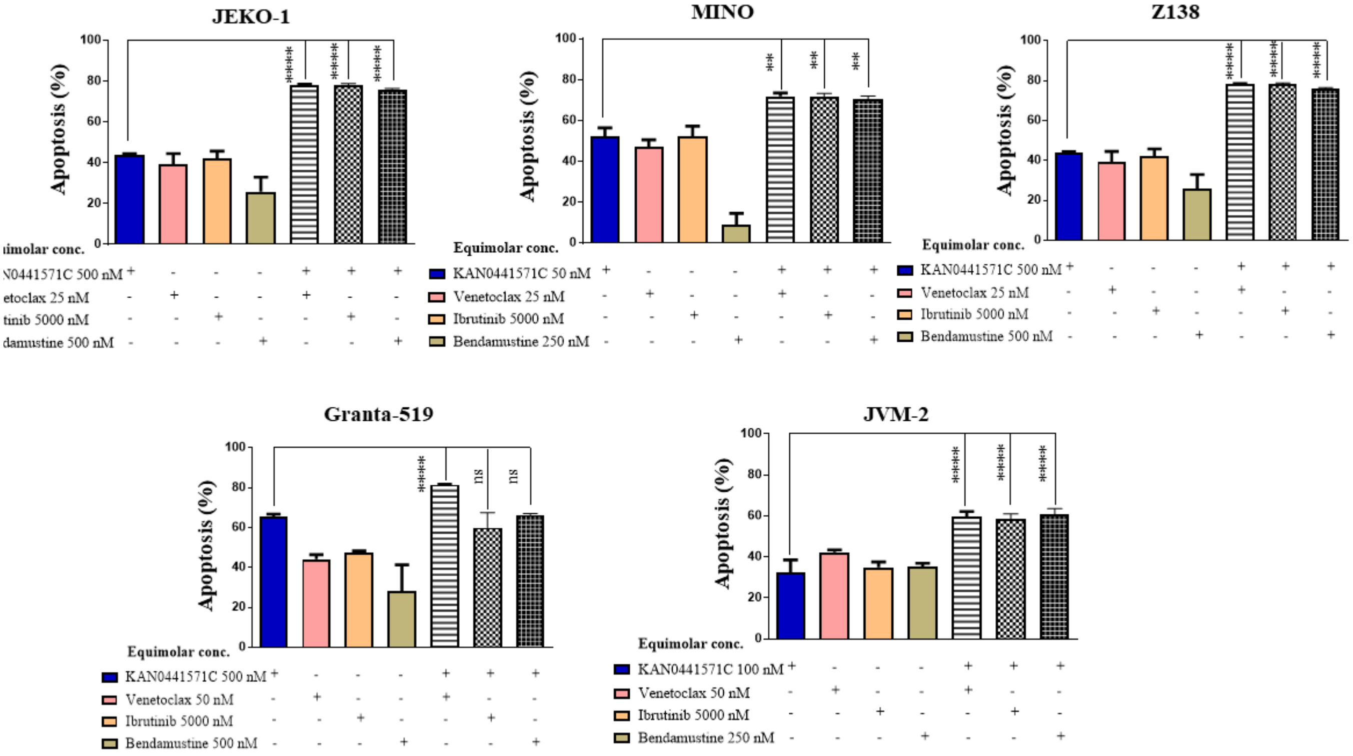

Supplementary Figure S4: Western blots analysis of apoptotic proteins in patient derived MCL cells. Western blots for apoptotic proteins in patient derived MCL cells after incubation with KAN0441571C, venetoclax, ibrutinib, everolimus alone and in combinations as indicated at the top. The molecular weight of proteins is shown fir each blot (A). Relative density of each protein band to beta actin (B).

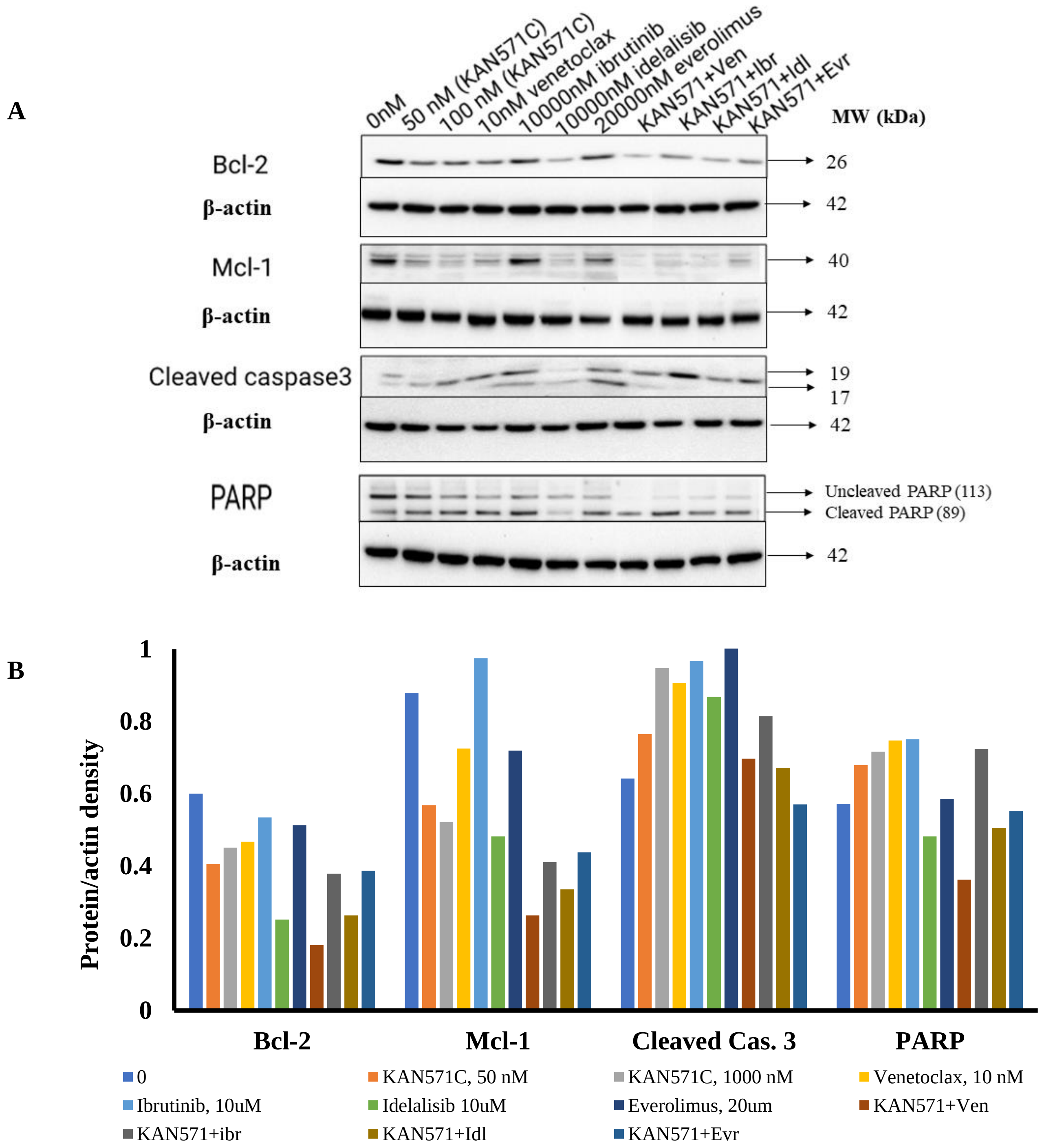

Supplementary Figure S5: Effects of KAN0441571C, venetoclax, ibrutinib, idelalisib, everolimus alone and in combinations on ROR1 and intracellular signaling molecules. Western blots of total and phosphorylated proteins after incubation with KAN0441571C, venetoclax, ibrutinib, idelalisib, everolimus alone and in combinations as indicated at the top (A). Densitometry analysis of pROR1/ROR1, pAKT/AKT, pPI3K/PI3K and, pBTK/BTK (B).

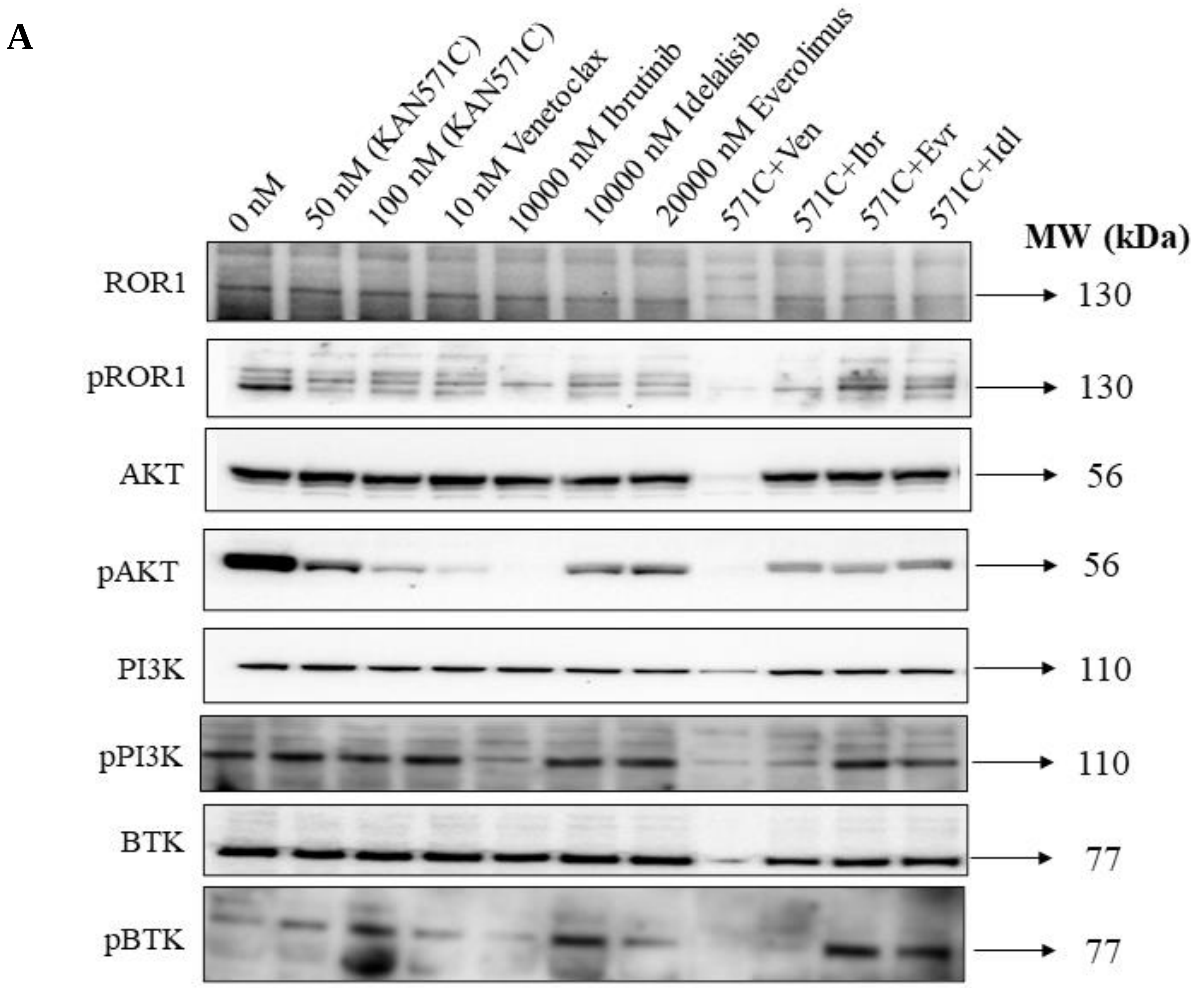

B

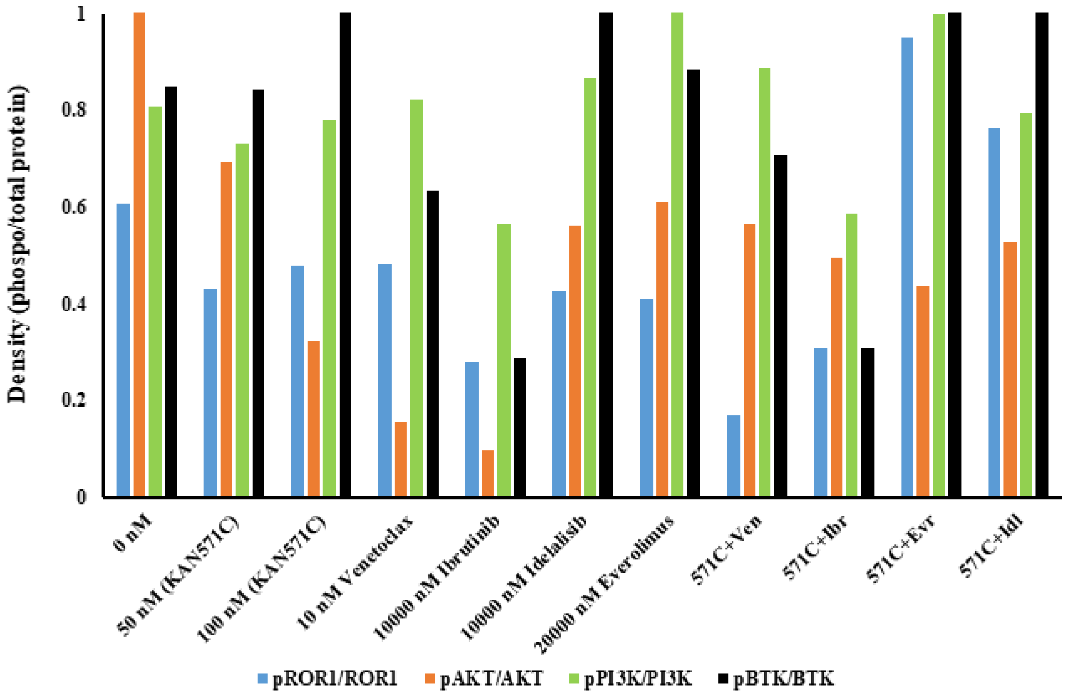
Density (phospo/total protein)
1
0.8
0.6
0.4
0.2
0
0 nM
50 nM (KAN571C)
100 nM (KAN571C)
10 nM Venetoclax
10000 nM Ibrutinib
10000 nM Idelalisib
20000 nM Everolimus
571C+Ven
571C+Ibr
571C+Evr
571C+Idl
pROR1/ROR1
pAKT/AKT
pPI3K/PI3K
pBTK/BTK

Supplementary Figure S6: Induction of apoptosis (flowcytometry Annexin/PI) in primary MCL cells. Representative figures of patients are shown in (A) (2013#020); (B) (2010#040) and (C) (2013#058) using a combination of KAN0441571C (25 nM) and venetoclax (5 nM). Untreated or DMSO (10 ul/ml) were used as controls (24 h). Induction of apoptosis (48 h) in the MCL cell line MINO (D) using various conc. of KAN0441571C (25-2000 nM) and DMSO (10 ul/ml) as a negative control is also shown

A

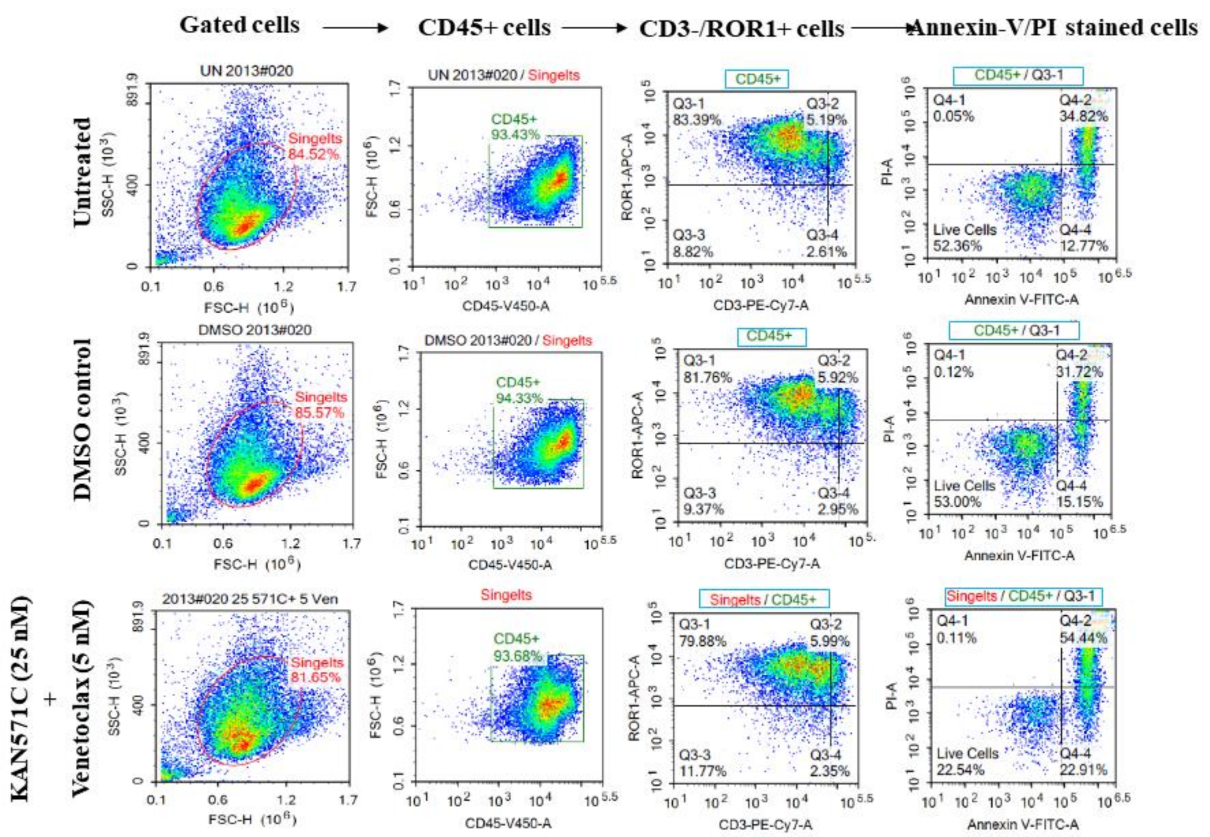

B

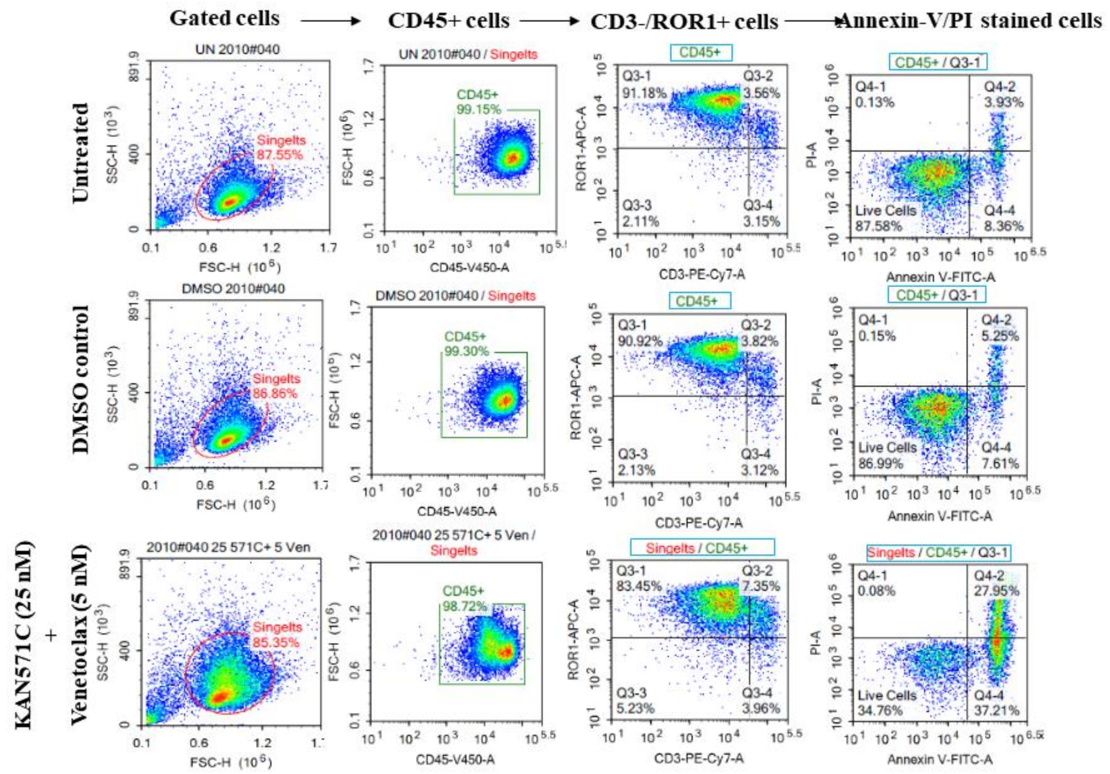
Gated cells → CD45+ cells → CD3-/ROR1+ cells → Annexin-V/PI stained cells
Untreated
DMSO control
KAN571C (25 nM) + Venetoclax (5 nM)
UN 2010#040
Singelts 87.55%
UN 2010#040 / Singelts
CD45+ 99.15%
CD45+
Q3-1 91.18%
Q3-2 3.56%
Q3-3 2.11%
Q3-4 3.15%
CD45+ / Q3-1
Q4-1 0.13%
Q4-2 3.93%
Live Cells 87.58%
Q4-4 8.36%
DMSO 2010#040
Singelts 86.86%
DMSO 2010#040 / Singelts
CD45+ 99.30%
Q3-1 90.92%
Q3-2 3.82%
Q3-3 2.13%
Q3-4 3.12%
Q4-1 0.15%
Q4-2 5.25%
Live Cells 86.99%
Q4-4 7.61%
2010#040 25 571C+ 5 Ven
Singelts 85.35%
2010#040 25 571C+ 5 Ven / Singelts
CD45+ 98.72%
Singelts / CD45+
Q3-1 83.45%
Q3-2 7.35%
Q3-3 5.23%
Q3-4 3.96%
Singelts / CD45+ / Q3-1
Q4-1 0.08%
Q4-2 27.95%
Live Cells 34.76%
Q4-4 37.21%
SSC-H (10^3)
FSC-H (10^6)
CD45-V450-A
ROR1-APC-A
CD3-PE-Cy7-A
PI-A
Annexin V-FITC-A

C

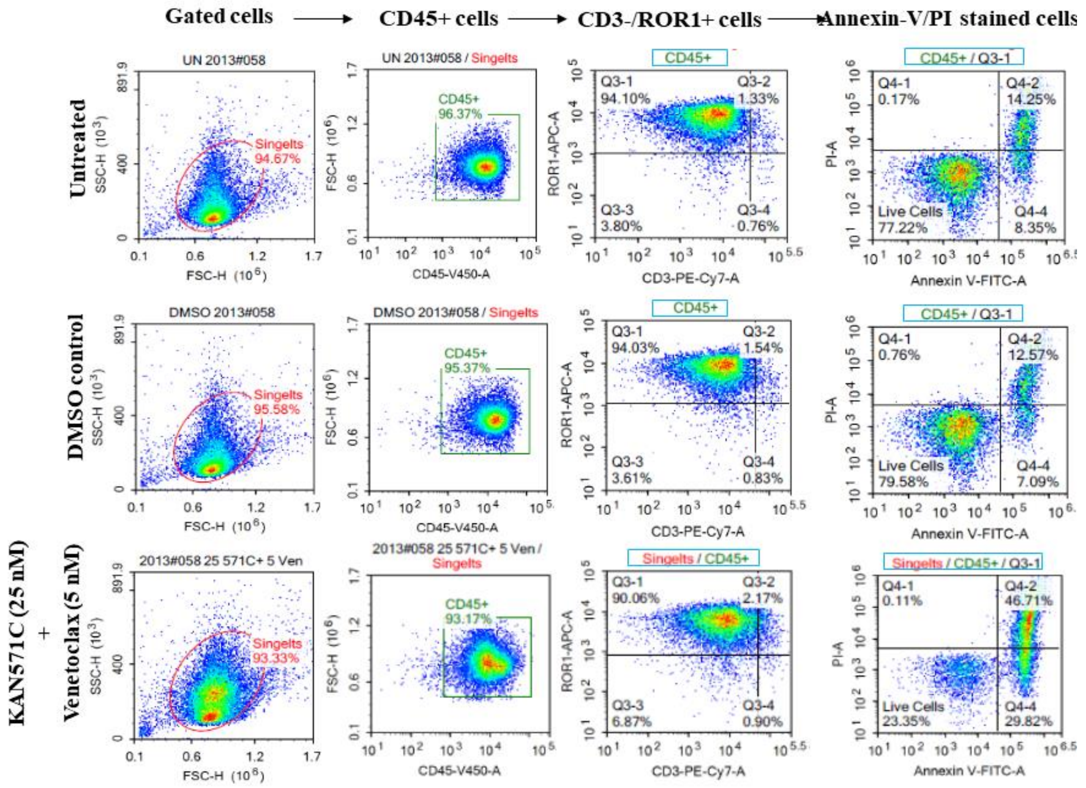
Gated cells → CD45+ cells → CD3-/ROR1+ cells → Annexin-V/PI stained cells
Untreated
DMSO control
KAN571C (25 nM) + Venetoclax (5 nM)
UN 2013#058
Singelts 94.67%
UN 2013#058 / Singelts
CD45+ 96.37%
CD45+
Q3-1 94.10%
Q3-2 1.33%
Q3-3 3.80%
Q3-4 0.76%
CD45+ / Q3-1
Q4-1 0.17%
Q4-2 14.25%
Live Cells 77.22%
Q4-4 8.35%
DMSO 2013#058
Singelts 95.58%
DMSO 2013#058 / Singelts
CD45+ 95.37%
Q3-1 94.03%
Q3-2 1.54%
Q3-3 3.61%
Q3-4 0.83%
Q4-1 0.76%
Q4-2 12.57%
Live Cells 79.58%
Q4-4 7.09%
2013#058 25 571C+ 5 Ven
Singelts 93.33%
2013#058 25 571C+ 5 Ven / Singelts
CD45+ 93.17%
Singelts / CD45+
Q3-1 90.06%
Q3-2 2.17%
Q3-3 6.87%
Q3-4 0.90%
Singelts / CD45+ / Q3-1
Q4-1 0.11%
Q4-2 46.71%
Live Cells 23.35%
Q4-4 29.82%
SSC-H (10^3)
FSC-H (10^6)
CD45-V450-A
ROR1-APC-A
CD3-PE-Cy7-A
PI-A
Annexin V-FITC-A

D

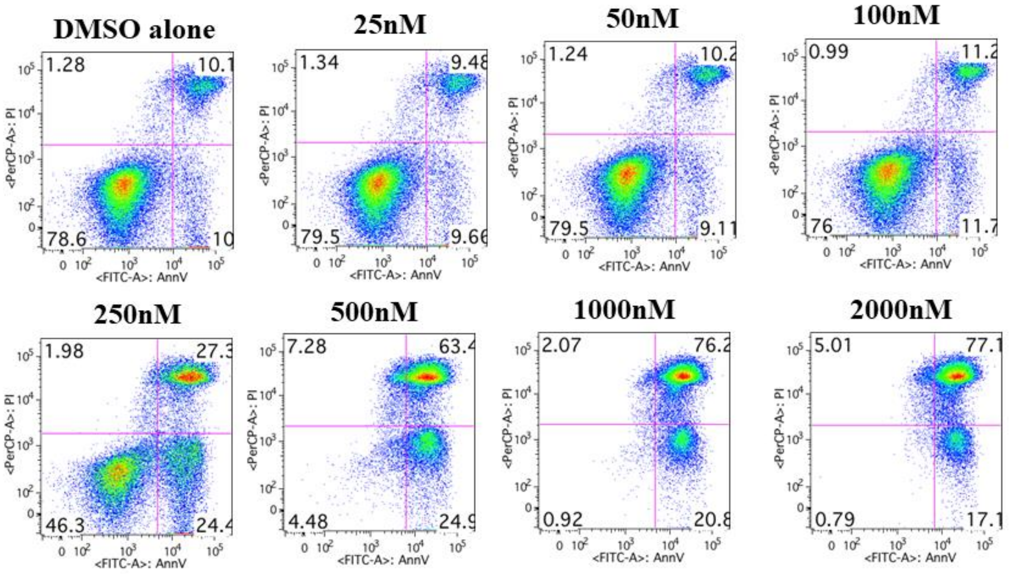
DMSO alone
1.28
10.1
78.6
10
25nM
1.34
9.48
79.5
9.66
50nM
1.24
10.2
79.5
9.11
100nM
0.99
11.2
76
11.7
250nM
1.98
27.3
46.3
24.4
500nM
7.28
63.4
4.48
24.9
1000nM
2.07
76.2
0.92
20.8
2000nM
5.01
77.1
0.79
17.1
<PerCP-A>: PI
<FITC-A>: AnnV
